# Resistance Status of Bacteria from a Health Facility in Ghana: A Retrospective Study

**DOI:** 10.1155/2021/6648247

**Published:** 2021-03-04

**Authors:** Abass Inusah, Elvis Quansah, Kwabena Fosu, Isaac Dadzie

**Affiliations:** ^1^Department of Medical Laboratory Science, School of Allied Health Sciences, University of Cape Coast, Cape Coast, Ghana; ^2^Microbiology Department, Bolgatanga Regional Hospital, Bolgatanga, Ghana; ^3^Department of Microbiology and Immunology, School of Medical Science, University of Cape Coast, Cape Coast, Ghana

## Abstract

**Background:**

Regardless of the global concerted effort to control the development and spread of antimicrobial resistance, increasing cases are continually documented at many medical centres. This situation is reinforced by inadequate information on the trend of resistance resulting from lack of regular antimicrobial resistance surveillance. The present study sought to detect the number of multidrug-resistant (MDR), extended drug-resistant (XDR), and pandrug-resistant (PDR) bacterial isolates at a health facility in Ghana from January 2018 to July 2020.

**Method:**

A total of 800 data on antimicrobial testing results were extracted from the records of the health facility. The extracted data were explored for the detection of MDR, XDR, and PDR. The study further determined the use of antibiotics using the multiple-drug resistance index (MDRI).

**Results:**

Except for *Staphylococcus* and *Neisseria* spp., all bacterial isolates showed extremely high (100%) proportion of MDR. Although only *Staphylococcus* spp. (38 (4.8%)) was observed to be XDR, the rest of the bacteria showed the potential to attain the status of XDR or PDR. MDRI indicated high use of antibiotics in the health facility.

**Conclusion:**

The high antimicrobial resistance observed by the study underscores the need for prompt and effective antibiotic resistance control strategies.

## 1. Background

Antibiotics constitute one of the most routinely used medications in clinical settings. In Ghana, recent evidence shows more than half (51.4%) of admitted patients receive one or more antibiotics [1]. A recent study revealed that about 65% of pregnant women are administered antibiotics at some stage during pregnancy [2]. Nonetheless, there have been growing reports of high levels of antibiotic resistance and emerging resistance mechanisms in many health facilities in Ghana [3, 4]—it is thought to be particularly high in the northern part [5].

The growing menace of antibiotic resistance (AMR) is more worrying against the backdrop of limited discovery of new antibiotics [6]. In fact, AMR remains one of the top priorities for the World Health Organization following the increasing antibiotic treatment failures recorded in many health facilities worldwide [7, 8]. Its potential to derail the progress of modern medicine—treatment and management of debilitating medical conditions including cancer, organ transplantation, diabetes, and caesarean sections—is a cause for serious concern [8]. The problem is attributed to the inappropriate and indiscriminate use of antibiotics which engenders a gradual evolution of multidrug resistance (MDR), extended drug resistance (XDR), and pandrug resistance (PDR) microbes. Skipping of the antibiotic regimen and suboptimal medication exert drug pressure on target bacteria, which select for the resistant (MDR, XDR, and PDR) subpopulations. Subsequent clonal expansion and horizontal resistant gene transfer ensure a complete, if not near complete, ineffectiveness of the frequently used antibiotics.

A myriad of studies have shown that bacteria exhibiting MDR, XDR, and PDR are the principal organisms that undermine successful treatment of nosocomial infections [9–11]. Lack of information on the resistant trend of these “superbugs” ensures their continual evolution over time and also retards appropriate control strategies in any case [12]. In light of this, there is the need for every health facility to periodically assess antibiotic use and understand the trend of resistance. The extrapolation of the multiple-drug resistance index (MDRI) has long been one of the most effective tools used to assess the overuse of antibiotics in health facilities. MDRI simplifies effective communication of antimicrobial resistance to policymakers and nonexperts and thereby facilitates informed and concerted response [13].

The present study retrospectively evaluates the trend of antimicrobial resistance by detecting MDR, XDR, and PDR in clinically recovered bacterial isolates at Bolgatanga Regional Hospital, Ghana.

## 2. Method

### 2.1. Study Area

The study was carried out at Bolgatanga Regional hospital, Ghana. The facility serves as a referral hospital to all the district health centres within the Upper East Region of Ghana and other health facilities situated at Ghana's border with Burkina Faso and the Republic of Togo. Recent expansion has increased the total bed capacity of the facility to 556.

### 2.2. Study Design

This study is a retrospective analysis of routine recovered bacterial isolates subjected against a panel of antibiotics for susceptibility testing. The study spun from January 2018 to July 2020. Records were gathered from May 2020 to July 2020.

### 2.3. Data Extraction

The data extracted from the health facility's record included the type of specimens, year of bacteria isolation, names of the pathogens, and the results of antibiotic susceptibility testing. Data of positive bacterial culture with susceptibility test records were included. Records that showed no bacteria growth were excluded. All information was confirmed by all the microbiology laboratory staff and reconfirmed by the microbiology unit supervisor and the chief biomedical scientist.

### 2.4. Collection and Identification of Clinical Isolates

The laboratory recovered bacterial isolates from varying specimens including urine, blood, sputum, seminal fluid, aspirate, cerebrospinal fluid, and swabs from various body sites (vagina, ear, throat, urethral, and wound). Gram staining and morphological identification were done following procedures previously described [14]. A set of biochemical tests including coagulase and catalase (for Gram-positive cocci), lactose fermentation, indole, citrate utilization, urease, triple sugar iron reaction, and oxidase test were performed by the laboratory.

### 2.5. Antimicrobial Susceptibility Test

The laboratory performed antimicrobial susceptibility testing (AST) on Mueller–Hinton agar (Oxoid, England) using Kirby–Bauer disc diffusion method and interpreted in accordance with the clinical laboratory guidelines [15]. AST was performed using a total of 22 antibiotics, ampicillin (10 *µ*g), tetracycline (30 *µ*g), cotrimoxazole (25 *µ*g), gentamicin (10 *µ*g), cefuroxime (30 *µ*g), vancomycin (30 *µ*g), chloramphenicol (10 *µ*g), ceftriaxone (30 *µ*g), cefotaxime (30 *µ*g), ciprofloxacin (30 *µ*g), amikacin (30 *µ*g), meropenem (10 *µ*g), Augmentin (30 *µ*g), piperacillin (100 *µ*g), nitrofurantoin (300 *µ*g), nalidixic acid (30 *µ*g), ceftazidime (30 *µ*g), norfloxacin (30 *µ*g), levofloxacin (5 *µ*g), penicillin (1.5 *µ*g), cloxacillin (5 *µ*g), and erythromycin (5 *µ*g), manufactured by Biomark Laboratories, Pune, India. The resistance patterns of the isolates were explored for MDR, XDR, and PDR using the consensus definition expounded by Magiorakos et al. [16]. The 22 antibiotics' discs were grouped into 12 classes (penicillin, aminoglycosides, cephalosporins, fluoroquinolones, tetracyclines, sulfamethoxazole/trimethoprim, chloramphenicols, nitrofurantoin, vancomycin, carbapenem, beta-lactam/beta-lactam inhibitors, and macrolides) using the “2019 WHO AWaRe classification of antibiotics for evaluating and monitoring.” [17] MDR isolates were identified as isolates that were resistant to at least one agent in three or more antimicrobial categories. XDR isolates were defined as isolates resistant to at least one agent in all but two or fewer antimicrobial categories. PDR isolates were identified as isolates that were resistant to all agents in all antimicrobial categories. MDRI was extrapolated as the ratio of the number of antibiotics to which the isolates showed resistance to the total number of antibiotics against which the isolates were tested. A cutoff >0.2 was used as an indicator for high-level use of antibiotics, whereas a cutoff ≤0.2 indicates low-level use of antibiotics [18].

### 2.6. Data Analysis

Analysis was done with the assumption of unequal variance due to unequal sample sizes of the indexed variables. Mann–Whitney test was used to compare two independent continuous variables. Analysis was done with IBM SPSS statistical software (version 21). Graphs were generated using GraphPad Prism 8.0.2. All analyses were done at an alpha value of 0.05.

## 3. Results

A total of 800 bacterial isolates were included in the study of which 473 (59.1%) and 327 (40.9%) were Gram negative and Gram positive, respectively (Table 1). The total isolates for 2018, 2019, and 2020 were 360, 290, and 150, respectively. The most prevalent isolate was *Staphylococcus* spp. (320 (40.0%)) followed by *Escherichia coli* (203 (25.4%)), *Pseudomonas* spp. (118 (14.8)), *Klebsiella* spp. (90 (11.4%)), and *Proteus* spp. (28 (3.5%)). The isolates were recovered from aspirate (5 (0.6%)), blood (54 (6.8%)), cerebrospinal fluid (12 (1.5%)), ear swab (8 (1.0%)), high vaginal swab (191 (23.9%)), semen (5 (0.6%)), sputum (56 (7.0%)), stool (4 (0.5%)), throat swab (4 (0.5%)), urethral swab (15 (1.9%)), urine (254 (31.8)), and wound swab (192 (24.0%)).

**Figure 1:**
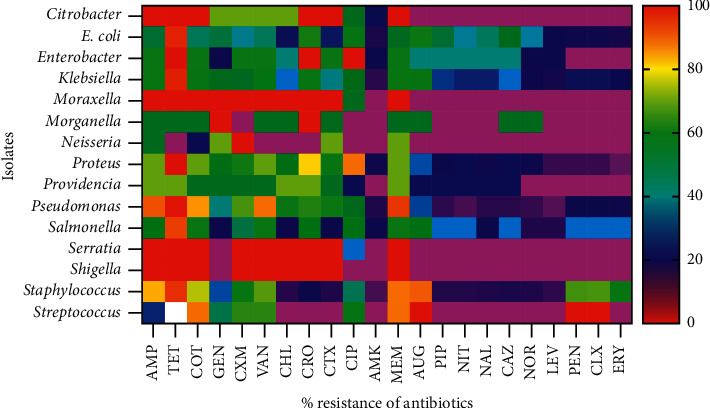
Heat map showing phenotypic antibiotic resistance pattern of bacteria at Bolgatanga Regional Hospital, January 2018–July 2020.

The phenotypic resistance traits of the isolates are presented in the heat map shown in Figure 1. From the results, high resistant percentages (>90%; in red) were observed for *Citrobacter* spp., *Moraxella* spp., *Serratia* spp., and *Shigella* spp. against ampicillin, tetracycline, cotrimoxazole, cefuroxime, chloramphenicol, ceftriaxone, and ceftazidime. From the result, <80% of the most prevalent isolates (PI), (*E. coli, Klebsiella* spp., *Pseudomonas* spp., *Proteus* spp., and *Staphylococcus* spp.) showed resistance against tetracycline. The PIs showed >60% resistance against meropenem and >40 resistance against the inexpensive antibiotics (tetracycline, cotrimoxazole, cefuroxime, and chloramphenicol). Approximately 90% of *Pseudomonas* was resistant to meropenem. Noticeably, amikacin (AMK) appeared to be the most potent antibiotic against all the isolates. The proportion of *Staphylococcus* and *Streptococcus* spp. resistant against vancomycin was 74% and 71%, respectively.

Overall proportions of 796 (99.6%), 38 (11.9%), and 0(0.0%) were recorded for MDR, XDR, and PDR isolates, respectively. Except for *Neisseria* (3 (75%)) and *Staphylococcus* (318 (99.4%)), all the bacterial isolates exhibited 100% MDR against the 12 classes of antibiotics (Table 2). Only *Staphylococcus* (38 (11.9%)) isolates appeared to show extended drug resistance status. No isolate produced a pandrug resistance status.

**Figure 2:**
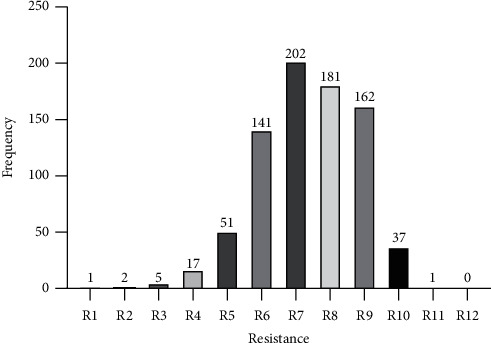
Multidrug resistance pattern of bacterial isolates from Bolgatanga Regional Hospital, Ghana, January 2018–July 2020.

Of the total 800 isolates, high proportions of 141 (17.6%), 202 (25.3%), 181 (22.6%), 162 (20.3%) were found to be resistant to at least one antibiotic in 6, 7, 8, and 9 different classes of antibiotics, respectively. One *Staphylococcus* isolate was found to be resistant to at least one antibiotic in all 12 antibiotic classes but one. The frequency distribution of the resistance status of the isolates is shown in Figure 2.

The MDR statuses for 2018 and 2019 were 360 (100%) and 290 (100%) and 197 (98.0%). The proportion of XDR bacterial isolates was highest in 2019 (15 (5.4%)) followed by 2020 (7 (4.7%)) and 2018 (16 (4.4%)). The distribution of MDR, XDR, and PDR is shown in Table 3.

The average multiple-drug resistance index for 2018, 2019, and 2020 was 0.45 (0.20–0.90), 0.45 (0.2–0.50), and 0.42 (0.0–0.5), respectively. From Table 4, all the isolates for 2018 and 2019 had MDRI index >0.2. Only a relatively small proportion (5 (3.3%)) of isolates had MDRI <0.2 of which all were *Staphylococcus* spp. recovered in 2020.

The MDRI for Gram-negative and Gram-positive isolates was 0.43 (0.10–0.50) and 0.45 (0.0–0.90). Mann–Whitney test showed no significant (*P* value >0.05) association between the MDRI for Gram-negative isolates (0.431) and Gram-positive isolates (0.430). Noticeably, 1 (0.3) of the isolates in 2018 had a MDRI of 0.9.

## 4. Discussion

Owing to the existing research data on antibiotic resistance available in Ghana [4, 19–23], it appears that health facilities in the northern zone are disproportionately disadvantaged resulting in the sparsity of empirical evidence needed for local and regional action. Surprisingly, a large amount of quality data is generated by health facilities in the northern part of Ghana but remain underutilized due to lack of interest. To understand the resistance pattern and status of bacterial isolates from Bolgatanga Regional Hospital, secondary data covering a total of 800 bacteria isolates were collected and evaluated. The data revealed that high proportions of Gram-negative isolates relative to Gram-positive isolates were involved in clinical infections frequently recorded at Bolgatanga Regional Hospital. Notably, *E. coli* and *Staphylococcus* spp. were the most prevalent Gram-negative and Gram-positive isolates, respectively, as observed in many previous studies in Ghana and elsewhere [24, 25].

Overall, the isolates were highly resistant to ampicillin, tetracycline, cotrimoxazole, cefuroxime, chloramphenicol, ceftriaxone, and ceftazidime. The foregoing antibiotics are from five different classes of antibiotics: penicillins, tetracyclines, sulfamethoxazole/trimethoprim, chloramphenicols, and cephalosporins, which suggest wide-range resistance capabilities of the bacterial isolates. In particular, the frequently isolated bacteria, *Staphylococcus* spp., *E. coli, Klebsiella* spp*., Pseudomonas* spp., and *Proteus* spp., showed high (>40%) resistance to the generally inexpensive broad-spectrum antibiotics: tetracycline, cotrimoxazole, cefuroxime, and chloramphenicol; this observation concurs with findings from a number of previous studies in Ghana [5, 25, 26]. On the contrary, amikacin was found to be the most effective antibiotic agent against all bacterial species—both Gram-negative and Gram-positive isolates. This finding corroborates the findings that showed amikacin as the most effective antibiotic agent against clinical isolates in Ghana [5, 27]. The high-resistance pattern of bacterial isolates from Bolgatanga Regional Hospital could be explained by its geographical location as previously explained [5].

A myriad of studies affirm that patients infected with MDR bacteria often have prolonged hospital stay and poor prognosis [28, 29]. This outcome is even worse in patients infected with XDR and PDR isolates. In this present study, except for *Neisseria* spp. and *Staphylococcus* spp., all the isolates were found to be 100% multidrug resistant. The high proportion of MDR isolates recorded in this present study is aberrant to an earlier study that reported lower proportions (11.8%–78.7%) of multidrug resistance bacteria at eleven hospitals in Ghana [25]. Paradox to the 0.0% PDR bacteria reported by this present study, a proportion of PDR clinical bacterial isolates have been documented at a medical center in Ethiopia [30]. The difference in study sites, time of study, and the subtle difference in bacterial species and number could argue for the disparities observed between these studies. Among the Gram-positive cocci, comparable XDR proportions of *Staphylococcus* (38 (15.1%)) and *Streptococcus* (0 (0.0%)) were observed as reported in India [10].

Although only *Staphylococcus* spp. was found to be XDR, worryingly, high proportions (686 (85.5%)) of the isolates were resistant to at least one antibiotic from 6, 7, 8, and 9 different categories of antibiotics (Figure 2). Against this finding, there is an ominous likelihood of MDR isolates to traverse into XDR or PDR “superbugs.” Accordingly, prompt and effective control strategies at Bolgatanga Regional Hospital would be required to prevent any quantum leap from MDR to XDR or PDR. The relative high resistance proportion of *Staphylococcus* spp. observed in this present study could be attributed to its notorious ability to accumulate enormous antibiotic-resistant determinants.

The present study further determined the trend of use or disuse of antibiotics using MDRI. This was to provide a clear view on the extent of antibiotic pressure in the environment within which the bacterial isolates thrived. Results from this present study showed that all bacterial species in 2018 and 2019 had MDRI >0.2, whereas only 5 (3.3%) bacterial species in 2020 had MDRI <0.2. The MDRI somewhat concludes that nearly all the bacterial isolates originated from an environment where there is high use of antibiotics.

It is worthwhile to note that the overall prevalence of XDR should be interpreted with caution as *Staphylococcus* spp. was the only composite isolate. The MDRI should also be interpreted with caution as it was possibly influenced by the high proportions of resistant *Staphylococcus* spp. as described earlier [31].

## 5. Conclusion

Against the findings of this present study, there were high-resistant bacterial isolates at Bolgatanga Regional Hospital, January 2018–July 2020. Resistance in *Staphylococcus* spp. was high. Also, there are high levels of MDR isolates which are likely to attain XDR or PDR status if immediate action is not taken. Finally, the findings suggest that there is high use of antibiotics at Bolgatanga Regional Hospital, and as such, it is important to critically evaluate and continually track the use of antibiotics to ensure sustained efficacies of the routinely used antibiotics.

## Figures and Tables

**Table 1:** Frequency of bacterial isolates from Bolgatanga Regional Hospital, January 2018–July 2020.

Isolate	2018	2019	2020	Total
*Gram negative*				
*E. coli*	103 (28.6%)	70 (21.4%)	30 (20.0%)	203 (25.4%)
*Klebsiella* spp.	52 (14.4%)	26 (9.0%)	12 (8.0%)	90 (11.4%)
*Proteus* spp.	5 (1.4%)	17 (5.9%)	6 (4.0%)	28 (3.5%)
*Pseudomonas* spp.	60 (16.7%)	49 (16.9%)	9 (6.0%)	118 (14.8)
*Citrobacter* spp.	0 (0.0%)	3 (1.0%)	1 (0.7%)	4 (0.5%)
*Enterobacter* spp.	0 (0.0%)	9 (1.4%)	4 (0.7%)	5 (0.6%)
*Moraxella* spp.	0 (0.0%)	2 (0.7%)	0 (0.0%)	2 (0.3%)
*Morganella* spp.	0 (0.0%)	2 (0.7%)	0 (0.0%)	2 (0.3%)
*Providencia* spp.	0 (0.0%)	2 (0.7%)	2 (1.3%)	4 (0.5%)
*Salmonella* spp.	5 (1.4%)	3 (1.0%)	1 (0.7%)	9 (1.1%)
*Serratia* spp.	0 (0.0%)	3 (1.0%)	0 (0.0%)	3 (0.4%)
*Shigella* spp.	0 (0.0%)	1 (0.3%)	0 (0.0%)	1 (0.1)
*Neisseria* spp.	0 (0.0%)	0 (0.0%)	4 (2.7%)	4 (0.5%)

*Gram positive*				
*Staphylococcus* spp.	135 (37.5%)	106 (36.6%)	99 (52.7)	320 (40.0%)
*Streptococcus* spp.	0 (0.0%)	2 (0.7%)	5 (3.3%)	7 (0.0%)

Total	360 (100%)	290 (100%)	150 (100%)	800 (100%)

**Table 2:** Resistance profile of bacterial isolates from Bolgatanga Regional Hospital, January 2018–July 2020.

Isolate	Resistance status (*n* (%))		
	MDR	XDR	PDR
*Gram negative*			
*E. coli*	203 (100%)	0 (0%)	0 (0%)
*Klebsiella* spp.	90 (100%)	0 (0%)	0 (0%)
*Proteus* spp.	5 (100%)	0 (0%)	0 (0%)
*Pseudomonas* spp.	118 (00%)	0 (0%)	0 (0%)
*Citrobacter* spp.	4 (100%)	0 (0%)	0 (0%)
*Enterobacter* spp.	5 (100%)	0 (0%)	0 (0%)
*Moraxella* spp.	2 (100%)	0 (0%)	0 (0%)
*Morganella* spp.	2 (100%)	0 (0%)	0 (0%)
*Providencia* spp.	4 (100%)	0 (0%)	0 (0%)
*Salmonella* spp.	9 (100%)	0 (0%)	0 (0%)
*Serratia* spp.	3 (100%)	0 (0%)	0 (0%)
*Shigella* spp.	1 (100%)	0 (0%)	0 (0%)
*Neisseria* spp.	3 (75%)	0 (0%)	0 (0%)

*Gram positive*			
*Staphylococcus* spp.	318 (99.4%)	38 (11.9%)	0 (0%)
*Streptococcus* spp.	7 (100%)	0 (0%)	0 (0%)

Total	796 (99.6%)	38 (11.9%)	0 (0%)

MDR = multidrug resistance, XDR = extended drug resistance, and PDR = pandrug resistance.

**Table 3:** Drug resistance status of bacteria from Bolgatanga Regional Hospital stratified by years.

Year	Drug resistance status		
	MDR	XDR	PDR
2018	360 (100%)	16 (4.4%)	0 (0%)
2019	290 (100%)	15 (5.4%)	0 (0%)
2020	147 (98.0%)	7 (4.7)	0 (0%)
Total	797 (99.6%)	38 (4.8%)	0 (0%)

MDR = multidrug resistance, XDR = extended drug resistance, and PDR = pandrug resistance.

**Table 4:** Distribution of multiple-drug resistance indexes of bacterial isolates from 2018, 2019, and 2020 at Bolgatanga Regional Hospital.

MDRI	2018	2019	2020
0	0 (0%)	0 (0.0%)	1 (0.7%)
0.1	0 (0%)	0 (0.0%)	4 (2.7%)
0.2	3 (0.8%)	4 (1.4%)	7 (4.7%)
0.3	26 (7.2%)	24 (8.3%)	20 (13.3)
0.4	124 (34.4%)	88 (30.3%)	42 (28.0%)
0.5	206 (57.2%)	174 (60.0%)	76 (50.7%)
0.9	1 (0.3%)	0 (0.0%)	0 (0.0%)
Total	306 (100%)	290 (100%)	150 (100%)

MDRI = multiple-drug resistance index.

## Data Availability

All the data generated or analysed during this study are included within this published article.
